# Association between body mass index and diabetes mellitus in tuberculosis patients in China: a community based cross-sectional study

**DOI:** 10.1186/s12889-017-4101-6

**Published:** 2017-02-28

**Authors:** Jing Cai, Aiguo Ma, Qiuzhen Wang, Xiuxia Han, Shanliang Zhao, Yu Wang, Evert G. Schouten, Frans J. Kok

**Affiliations:** 1 The College of Public Health, Qingdao University, 38 Dengzhou Road, Qingdao, Shandong Province 266021 People’s Republic of China; 2 Linyi People’s Hospital, Linyi, 276000 China; 3 The Department of Public Health, Lanzhou University, 222 Tianshui South Road, Lanzhou, 730000 China; 4 Division of Human Nutrition, Wageningen University, Wageningen, 6700 The Netherlands

**Keywords:** Fasting plasma glucose, Receiver operating characteristic, Area under the curve

## Abstract

**Background:**

We planned to determine the association of body mass index (BMI) with diabetes mellitus (DM) and impaired fasting glucose (IFG) in Chinese pulmonary tuberculosis (PTB) patients.

**Methods:**

3,505 newly-diagnosed PTB patients registered in PTB clinics in Linyi of China between September 2010 and March 2013 were enrolled. DM and IFG were identified based on fasting plasma glucose levels. ROC analysis was used to predict the ability of screening of BMI for DM and IFG in PTB patients.

**Results:**

Compared with 18.5–23.9 kg/m^2^, patients with DM and IFG had significantly increased trends when BMI ≥ 24.0 kg/m^2^, and aORs were 2.28 (95%CI 1.44–3.60) and 1.30 (95%CI 1.04–1.64), respectively. After adjustment for age, gender, and educational level, there was an increased odd in BMI ≥ 23.41 kg/m^2^ for IFG, and a decreased odd in BMI < 19.82 kg/m^2^ for DM (*p* < 0.05). The cut-offs of BMI for screening IFG and DM in PTB patients were 22.22 kg/m^2^ (AUC 0.56) and 22.34 kg/m^2^ (AUC 0.59).

**Conclusions:**

In PTB patients, BMI is significantly associated with IFG and DM. However, the predictive power of BMI was not sufficient, so it may only be a limited screening tool for DM and IFG among PTB patients in China.

## Background

The co-morbidity of diabetes mellitus (DM) and pulmonary tuberculosis (PTB) represents a double burden with significant public health implications [1, 2]. Globally, although the incidence of PTB is slowly decreasing, an increase is seen in the proportion of PTB cases with DM. Moreover, the prevalence of DM steadily increases, especially in developing countries where PTB is highly endemic [3, 4]. Baker conducted a meta-analysis of observational studies about the association between DM and TB disease outcomes, and showed that DM was associated with worse treatment outcomes, and increased the risk of failure, death, and relapse among patients with PTB. Therefore, more attention should be paid to the control and prevention of PTB patients with DM [5].

Also, patients with impaired fasting glucose (IFG) are more prone to progress to the DM stage -approximately 8.8% per year in China [6, 7]- in the absence of interventional measures. In developing countries, the nutritional status has improved along with the economic growth. A number of studies have provided strong evidence of an association between patients who are overweight or obese and risk of DM [8–10]. The effect of BMI on DM has primarily focused on patients without other diseases. A low BMI is a significant individual risk factor for development of recent active TB [11], and patients who are overweight have a decreased incidence of TB [12]. Studies involving the association between BMI and DM or IFG in PTB patients are limited [13], and no data are available on using BMI cut-offs to predict DM or IFG in PTB patients.

We designed this cross-sectional study using primary data to explore the association of BMI with DM or IFG in PTB patients in China, and to determine the optimal BMI cut off value for prediction of DM or IFG in Chinese adult patients with PTB.

## Methods

### Study population

A total of 3,505 PTB patients, 18-45 years of age, were selected from counties of Linyi in Shandong province, China. All the PTB patients were newly-diagnosed and registered for Directly Observed Treatment, Short Course (DOTS) in PTB clinics between September 2010 and March 2013, and also were diagnosed in the PTB clinic of each county by X-ray and sputum smear examination. All participants have written informed consent.

### Detection of indices

DM was diagnosed by World Health Organization criteria based on the fasting plasma glucose (FPG) level (World Health Organization, 1999). The diagnosis of IFG was based on criteria for the classification of glucose tolerance based on the FPG level, which was defined as a FPG range from 5.6–6.9 mmol/L (American Diabetes Association, 2014). The participants fasted for 8–10 h before the blood testing.

Basic information and anthropometric indices of all patients were collected, such as age, gender, educational level, weight, and height. The BMI (kg/m^2^) was calculated using the following formula: BMI = weight (kg)/height (m)^2^. Underweight, normal weight, overweight, and obese categories were defined using the modified criteria for the Chinese population; the BMI cut-off values were 18.5 kg/m^2^, 24.0 kg/m^2^, and 28.0 kg/m^2^, respectively [14].

Blood lipids were also determined in fasting venous blood samples using an automatic biochemical analyzer in each clinic, and included triglycerides (TG), total cholesterol (TCHO), and high-density lipoprotein (HDL) levels.

### Statistical analysis

SPSS version 17.0 (SPSS, Inc. Chicago, IL) was used for statistical analyses. The characteristics of PTB patients in the DM, IFG and normal FPG groups and inter-quartile range (IQR) of BMI were compared and analyzed. The mean and standard deviation for continuous variables, such as age, BMI, and blood lipid content, and proportions for categorical variables, including the prevalence of DM or IFG, the percentage of age group, gender, and educational level, are reported. One-way analysis of variance was used to test continuous variables. A chi-square test was used to compare categorical variables. Multinomial logistic regression analyses were performed, and the variables for inclusion in the multivariate model were chosen based on plausibility and variables with *P* values <0.05 on univariate analysis were entered into the multivariate analysis. Receiver operating characteristic (ROC) analysis was used to predict the ability of diagnosis of BMI for DM and IFG in PTB patients. The sensitivity, specificity, positive predictive value (PPV), negative predictive value (NPV), positive likelihood ratio (PLR), negative likelihood ratio (NLR) and Youden’s index were used for ROC analysis. Youden’s index is a single statistic that captures the performance of a dichotomous diagnostic test. It was defined for all points of the ROC curve, and the maximum value of the index was used as a criterion for selecting the optimum cut-off point. Youden’s index = Sensitivity + Specificity – 1. All variables were checked for collinearity. Independent variables included age (divided into two categories [18–30 and 31–45 years]), gender, educational level, BMI (divided into three categories [<18.5 kg/m^2^ as underweight, 18.5–23.9 kg/m^2^ as normal weight, and ≥ 24.0 kg/m^2^ as overweight and obese]). A *P* value <0.05 was considered statistically significant.

## Results

### Descriptive data

A total of 3 505 PTB patients were included in our study (18–45 years of age), among whom 96 and 529 had co-existing DM and IFG, respectively. The mean age was 31 · 8 years for all PTB patients, and the mean age in the PTB + DM group was the highest (*P* < 0.05). In the PTB + IFG group, 65.2% were male, which was more than the PTB group (57.5%, *P* < 0.05). The percentage of patients with a high school or higher educational level in the PTB + DM and PTB + IFG groups were lower than the PTB group (*P* < 0.05). BMI means and the proportion of overweight or obese patients in the PTB + DM and PTB + IFG groups were higher than the PTB group (*P* < 0.05). The means of TG and TCHO were 1.6 mmol/L and 4.8 mmol/L in the PTB + DM group, respectively, which were higher than the PTB + IFG and PTB groups (*P* < 0.05).

Multivariate logistic regression analysis of DM or IFG in PTB patients is shown in Table 1. PTB patients in the younger age group and patients with a high educational level, had an odds ratio (OR) of the presence of DM and IFG that was significantly lower than 1.0 (*P* < 0.05). Overweight or obese patients had significantly higher odds ratios of DM and IFG (OR = 2.28, 95% confidence interval [CI] =1.44–3.60; OR = 1.30, 95% CI = 1.04–1.64, respectively). In addition, male gender was positively associated with the IFG (OR = 1.38, 95% CI = 1.14–1.68).

**Table 1:** Characteristics of PTB patients and odds ratios of having impaired fasting glucose (IFG) or diabetes mellitus (DM)

Variables	PTB + DM group (*n* = 96)			PTB + IFG group (*n* = 529)			PTB group (*n* = 2880) (ref)	*p*-value
	Mean ± SD/*N* (%)	OR (95% CI)	*P*-value	Mean ± SD/*N* (%)	OR (95% CI)	*P*-value	Mean ± SD/*N* (%)	
Age, years	36.39 ± 7. 0 * †	--	--	33.9 ± 8.0 *	--	--	31.2 ± 8.3	<0.001
Age group, *n* (%)								<0.001
18–30 years	21 (21.9)	0.26 (0.16–0.42)	<0.001	190 (35.9)	0.51 (0.42–0.62)	<0.001	1506 (52.3)	
31–45 years	75 (78.1)	1	--	339 (64.1)	1	--	1374 (47.7)	
Gender, *n* (%)								0.004
Male	56 (58.3)	1.03 (0.68–1.56)	0.876	345 (65.2)	1.38 (1.14–1.68)	0.001	1657 (57.5)	
Female	40 (41.7)	1	--	184 (34.8)	1	--	1223 (42.5)	
Education, *n* (%)								<0.001
High school or higher	16 (16.7)	0.19 (0.08–0.46)	<0.001	119 (22.5)	0.37 (0.24–0.58)	<0.001	892 (31.0)	
Primary or middle school	72 (75.0)	0.41 (0.19–0.87)	0.021	379 (71.6)	0.55 (0.36–0.85)	0.006	1902 (66.0)	
Illiteracy	8 (8.3)	1	--	31 (5.9)	1	--	86 (3.0)	
TG, mmol/L	1.6 ± 1.0 * †	--	--	1.3 ± 1.0	--	--	1.2 ± 1.0	<0.001
TCHO, mmol/L	4.8 ± 1.2 * †	--	--	4.4 ± 1.1	--	--	4.3 ± 1.3	0.002
HDL, mmol/L	1.7 ± 0.8	--	--	1.6 ± 0.7	--	--	1.5 ± 2.5	0.575
BMI, kg/m^2^	22.6 ± 3.4 *	--	--	22.2 ± 2.9 *	--	--	21.6 ± 2.8	<0.001
BMI group, *n* (%)								<0.001
Underweight	12 (12.5)	1.46 (0.77–2.76)	0.244	44 (8.3)	0.79 (0.56–1.10)	0.157	314 (10.9)	
Overweight or obese	30 (31.3)	2.28 (1.44–3.60)	<0.001	117 (22.1)	1.30 (1.04–1.64)	0.024	503 (17.5)	
Normal weight	54 (56.2)	1	--	368 (69.6)	1	--	2063 (71.6)	

* Compared with PTB group, *P* < 0.05; † Compared with PTB + IFG group, *P* < 0.05; -- The values could not be calculated; *SD* standard deviation, *ref* reference

### Prevalence of IFG or DM in PTB patients as a function of BMI quartiles

**Fig. 1:**
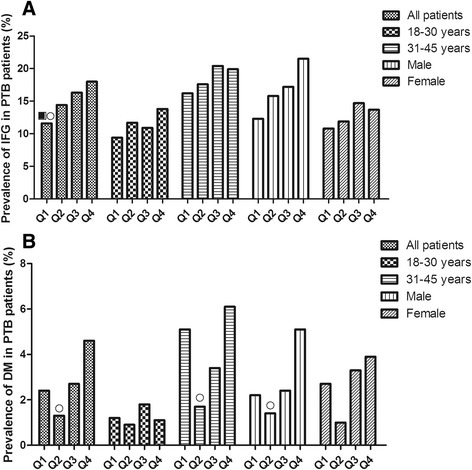
Prevalence of IFG or DM in PTB patients between BMI quartiles at different age and gender groups. **a** showed the prevalence of IFG in PTB patients between IQR of BMI at different age and gender groups. **b** showed the prevalence of IFG in PTB patients between IQR of BMI at different age and gender groups. ■ compared with Q3, *P* < 0.05; ○ compared with Q4, *P* < 0.05

The participants were divided into four groups based on the BMI baseline quartiles, as follows: Q1, <19.82 kg/m^2^; Q2, 19.82 kg/m^2^; Q3, 21.45 kg/m^2^; and Q4, 23.41 kg/m^2^ (Fig. 1). There was an increasing prevalence of IFG among the four groups (11.6%, 14.4%, 16.3%, and 18.0% respectively); in different age and gender categories, the prevalence of IFG was increased from Q1 to Q4. The prevalence of DM in the Q2 group was much lower than the Q4 group (*P* < 0.05); the prevalence in the patients 31–45 years of age and males were also the same trends.

**Fig. 2:**
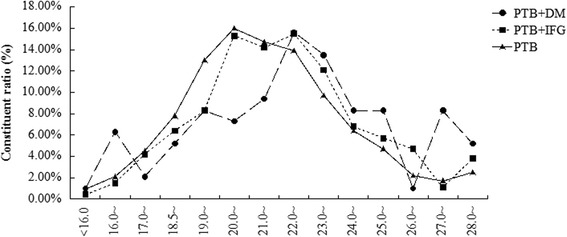
The constituent ratio of different BMI level among PTB + DM, PTB + IFG, PTB groups

The percentages of different BMI levels among the PTB + DM, PTB + IFG, and PTB groups are shown in Fig. 2. The distributions of the three groups were almost overlapping and all similar to normal distributions. At lower BMI (<21.0 kg/m^2^), the percentage in the PTB group was somewhat higher; at higher BMI, it was lower than the PTB + IFG group and PTB + DM group.

### Odds ratios of BMI quartiles for IFG or DM

The univariate and multivariate odds ratios for IFG and DM based on BMI quartiles are shown in Table 2. For IFG univariate ORs in Q3 and Q4 (model 1) were significantly greater than 1.0. After adjustment for age, gender, and educational level (model 4), only the OR in Q4 was still significant. For DM in model 1, the OR in Q4 was significantly greater than 1.0. After adjustment for age, gender, and educational level, only the OR in Q1 was significantly smaller than 1.0.

**Table 2:** Odds ratios of BMI quartiles for IFG or DM

		Q1 <19.82 kg/m^2^	Q2 19.82 kg/m^2^~	Q3 21.45 kg/m^2^~	Q4 23.41 kg/m^2^~
IFG	Model 1	1.00	1.27 (0.96–1.69)	2.49 (1.14–1.97) *	1.73 (1.32–2.27) *
	Model 2	1.00	1.15 (0.87–1.53)	1.28 (0.96–1.69)	1.37 (1.03–1.82) *
	Model 3	1.00	1.12 (0.84–1.48)	1.25 (0.94–1.65)	1.37 (1.03–1.81) *
	Model 4	1.00	1.12 (0.84–1.50)	1.27 (0.96–1.69)	1.41 (1.06–1.88) *
DM	Model 1	1.00	0.53 (0.25–1.11)	1.21 (0.67–2.19)	2.11 (1.23–3.61) *
	Model 2	1.00	0.43 (0.20–0.90) *	0.87 (0.47–1.59)	1.31 (0.75–2.31)
	Model 3	1.00	0.43 (0.20–0.90) *	0.86 (0.47–1.59)	1.31 (0.75–2.31)
	Model 4	1.00	0.44 (0.21–0.92) *	0.90 (0.49–1.66)	1.39 (0.79–2.46)

Model 1 unadjusted; Model 2 was adjusted by age; Model 3 was adjusted by Model 2 + gender; Model 4 was adjusted by Model 3 + educational level; *By multinomial logistic regression analyses, *P* < 0.05

### ROC of BMI for IFG or DM in PTB patients

The results of ROC analysis showed that BMI might be a weak potential predictor for IFG or DM in PTB patients (Table 3). With respect to IFG, the area under the ROC curve was 0.56 for all PTB patients and 0.57 for male patients. The optimal BMI cut-off for IFG was identified as 22.22 kg/m^2^ in all patients and 22.23 kg/m^2^ in males; and the PPV, NPV, PLR and NLR were 0.70, 0.38, 1.17 and 0.81 for all patients and 0.73, 0.38, 1.36 and 0.80 for males, respectively. With respect to DM, the area under the ROC curve was 0.59 for all PTB patients, 0.58 for patients 31–45 years of age, and 0.61 for males. The optimal BMI cut-off for DM was 22.34 kg/m^2^ in all patients, 22.53 kg/m^2^ in patients 31–45 years of age, and 22.41 kg/m^2^ in males; and the PPV, NPV, PLR and NLR were 0.77, 0.42, 1.58 and 0.65 for all patients, 0.78, 0.39, 1.45 and 0.64 for 31–45 years of age, 0.78, 0.43, 1.66 and 0.60 for males, respectively.

**Table 3:** Receiver Operator Curve analysis of the predictive value of BMI for impaired fasting glucose (IFG) and diabetes mellitus (DM) in categories of gender and age in PTB patients

		SE	AUC (95%CI)	*p*-value	Sensitivity	Specificity	PPV	NPV	PLR	NLR	Youden’s index	Associated criterion
IFG	Total	0.01	0.56 (0.53–0.58)	<0.001	0.61	0.48	0.70	0.38	1.17	0.81	0.09	22.22
	Age											
	18–30 years	0.02	0.54 (0.49–0.58)	0.104	--	--	--	--	--	--	--	--
	31–45 years	0.02	0.53 (0.49–0.56)	0.124	--	--	--	--	--	--	--	--
	Gender											
	Male	0.02	0.57 (0.54–0.60)	<0.001	0.49	0.64	0.73	0.38	1.36	0.80	0.13	22.23
	Female	0.02	0.53 (0.48–0.58)	0.188							--	--
DM	Total	0.03	0.59 (0.53–0.66)	0.002	0.60	0.62	0.77	0.42	1.58	0.65	0.22	22.34
	Age											
	18–30 years	0.07	0.50 (0.37–0.63)	0.977	--	--	--	--	--	--	--	--
	31–45 years	0.04	0.58 (0.50–0.65)	0.024	0.64	0.56	0.78	0.39	1.45	0.64	0.20	22.53
	Gender											
	Male	0.04	0.61 (0.53–0.70)	0.004	0.63	0.62	0.78	0.43	1.66	0.60	0.25	22.41
	Female	0.05	0.56 (0.46–0.67)	0.184	--	--	--	--	--	--	--	--

*PPV* positive predictive value, *NPV* negative predictive value, *PLR* positive likelihood ratio, *NLR* negative likelihood ratio, *SE* Standard Error, *AUC* area under the curve; Youden’s index = Sensitivity + Specificity − 1; -- When *p* > 0.05, there was no appropriate cut-off by ROC analysis, so the Youden’s index and associated criterion could not be shown

## Discussion

In this survey of Chinese adults (18–45 years of age), we showed that overweight and obesity were positively associated with DM and IFG in PTB patients. The prevalence of DM and IFG was higher when the BMI was ≥ 23.41 kg/m^2^ (Q4). Until now the optimal BMI cut off point for IFG and DM screening among PTB patients has not been estimated in China. Based on the current study, the optimal BMI cut-offs for predicting DM and IFG were 22.22 kg/m^2^ and 22.34 kg/m^2^, respectively.

After adjusted for the associated factors, including young age, high educational level, and excess weight, BMI was still a risk factor for DM or IFG in PTB patients. In a previous cohort study [15], the incidence of DM was mainly associated with overweight and obesity in China (28.3% among men and 31.3% among women). However, a inverse association and dose–response relationship between the incidence of TB and BMI up to 30.0 kg/m^2^ was shown in a previous meta-analysis [16]. The relative risk of TB in underweight patients (BMI < 18.5 kg/m^2^) compared to normal weight patients was estimated to be 3.2 (95% CI = 3.1–3.3) [17]. Meanwhile, an association between abnormal weight and elevated glucose (DM and IFG) may still exist. A survey administered in 49 developing countries showed that not only overweight, but also underweight might be involved in the pathogenesis of diabetes [18]. Thus, overweight and obesity in PTB patients should not be overlooked, whether or not it regards only a small proportion of PTB patients.

In the model adjusted by all of the relevant risk factors, a BMI in the 19.82–21.45 kg/m^2^ range was negatively associated with DM in PTB patients, and a BMI > 23.41 kg/m^2^ was positively associated with IFG. Moreover, for a BMI ≥ 23.41 kg/m^2^_,_ the prevalence of DM and IFG in PTB patients was much higher in our study. A cross-sectional study carried out in Spain showed that the prevalence of DM in overweight or obese patients was 23.6%, and the higher the BMI, the higher the prevalence of DM [19]. In the current study, some differences in the BMI associations still existed. Specifically, when the BMI was < 19.82 kg/m^2^ (Q1), the prevalence of DM in PTB patients presented slightly decreased, although the differences were not significant. This finding might be attributable to the impact of PTB. Abundant epidemiologic evidence has indicated that low BMI is a risk factor for PTB, and the biological mechanism underlying the relationship has been well-described by induced impairment of cellular immunity [20]. In addition, underweight patients might also be involved in the pathogenesis of DM through direct and indirect mechanisms [21]. Therefore, we should pay more attention to the factors associated with underweight, overweight, and obesity, who favor high prevalence of DM and IFG in PTB patients.

We were able to establish BMI cut-off values for DM and IFG in PTB patients in the current study. Although BMI might not be the strongest risk factor to screen undiagnosed DM compared with age, waist circumference, and a family history of DM [22], it is an easy to acquire anthropometric index for the prediction of DM. With respect to adults, the optimal BMI cut-off value for predicting the presence of DM was 23.3 kg/m^2^ for men and 24.0 kg/m^2^ for women [23]. A cross-sectional study conducted in northeast Chinese adults showed that combined with the waist-to-height ratio, a maximal BMI ≥ 23.0 kg/m^2^ for DM and ≥ 22.0 kg/m^2^ for glucose tolerance abnormalities were better anthropometric indices [24]. Also, the San Antonio Heart Cohort Study reported that BMI and waist circumference had equal power in predicting development of metabolic syndrome in non-Hispanic Whites and Mexican Americans [25]; thus, BMI is possibly an appropriate predictor for pathoglycemia. Of note, the optimal BMI cut-off points in our study were lower than other studies, which might because PTB patients were more likely to have a low BMI [11], and in young patients (18–45 years old), a lower BMI was more common [26].

There were some limitations in our study. First, there were some limitations in the generalizability of our findings: the age range and only Chinese participants. The age range in our survey was 18–45 years, which comprised young and middle-aged adults in China. Because there were no elderly in our study, which comprise a high-risk group and have a higher prevalence than young and middle-aged adults [27], the prevalence of DM and IFG would be low. And then, the results of this study were applied to Chinese population, so there might be different results in other countries. Second, Youden’s index was low, which meant the false-positive and false-negative values were high for BMI in predicting DM and IFG in PTB patients.

## Conclusions

In conclusion, the present study revealed that BMI might be a limited screening tool for DM and IFG in PTB patients in Chinese young and middle-aged people. However, the predictive power of BMI was still poor. This study is only a prelude to the upcoming research in co-existing infectious diseases and chronic non-communicable diseases, especially in Chinese populations. Further studies are warranted to verify the effects of nutritional status on the co-morbidity of these two diseases.

## Abbreviations

95% CI: 95% confidence interval
DM: Diabetes mellitus
DOTS: Directly Observed Treatment, Short Course
FPG: Fasting plasma glucose
HDL: High-density lipoprotein
IFG: Impaired fasting glucose
IQR: Inter-quartile range
NLR: Negative likelihood ratio
NPV: Negative predictive value
OR: Odds ratio
PLR: Positive likelihood ratio
PPV: Positive predictive value
PTB: Pulmonary tuberculosis
TCHO: Total cholesterol
TG: Triglycerides
